# Physical and Mechanical Properties of Hollow Fiber Membranes and Technological Parameters of the Gas Separation Process

**DOI:** 10.3390/membranes11080583

**Published:** 2021-07-30

**Authors:** Georgy Kagramanov, Vladimir Gurkin, Elena Farnosova

**Affiliations:** Faculty of Digital Technology and Chemical Engineering, Dmitry Mendeleev University of Chemical Technology of Russia, 125047 Moscow, Russia; wladimir0594@yandex.ru (V.G.); farelena@rambler.ru (E.F.)

**Keywords:** polymeric hollow-fiber membranes, gas separation, stress-strain state

## Abstract

The porous layer of composite and asymmetric hollow fiber membranes acts as a support and is exposed to strong mechanical stresses. The effect of external pressure on the polymer structure and, as a consequence, the separation characteristics of the membrane remains unsolved. Based on the solution of the Lamé approach to the calculation of the stress state of a hollow cylinder, a method of calculation was proposed for hollow fiber membranes. Calculations were based on the approximation of the isotropic nature of the physical and mechanical characteristics of the selective layer and substrate. Permissible deformation of the membrane’s selective layer was determined from the linear sector of strain-on-stress dependence, where Hooke’s law was performed. For these calculations, commercial polyethersulfone membranes were chosen with an inner and/or outer selective layer and with the following values of Young’s modulus of 2650 and 72 MPa for the selective and porous layers, respectively. The results obtained indicate that the dependence of the maximum allowable operating pressure on the substrate thickness asymptotically trends to a certain maximum value for a given membrane. Presented data showed that membranes with outer selective layer can be operated at higher working pressure. Optimal parameters for hollow fiber gas separation membrane systems should be realized, solving the optimization problem and taking into account the influence of operating, physicochemical and physicomechanical parameters on each other.

## 1. Introduction

One of the essential properties of polymers is the ability to change their structure, and, consequently, the physicochemical and mechanical properties. First of all, this is achieved by creating a certain molecular and supramolecular structure of the polymer, due to the appropriate synthesis conditions. When obtaining solid polymers and, accordingly, polymeric membranes with desired properties, it is necessary to ensure the structure formation of the polymeric material is obtained, i.e., to give to the macromolecules the desired shape and to achieve their definite interposition [1,2,3].

**Figure 1:**
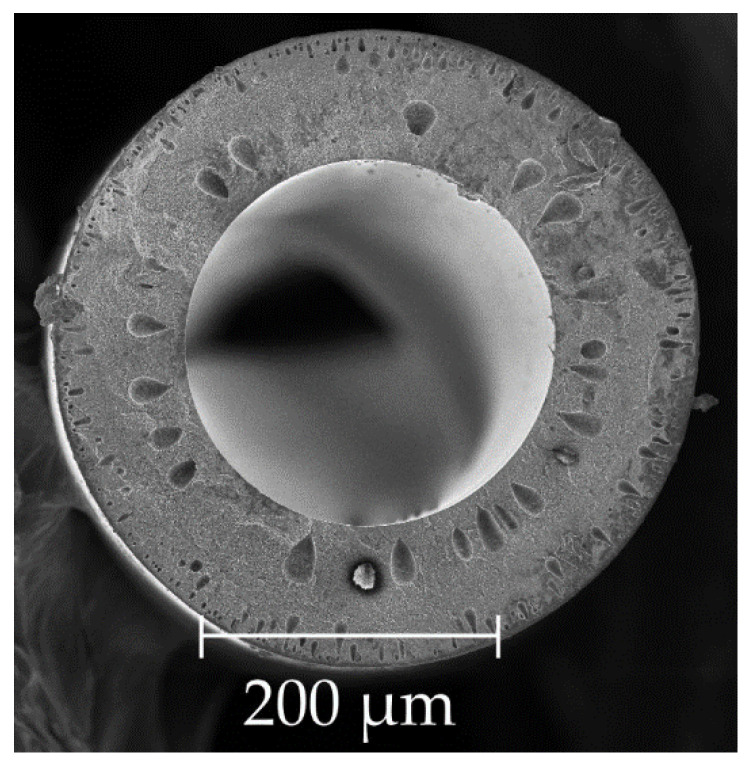
Photo of a hollow fiber [14].

Asymmetric pore size distribution in gas separation membranes is the most common type of their structure [4,5], which is characterized by the presence of a thin, dense non-porous surface layer and a porous sublayer with relatively large transport pores (Figure 1).

The substrates of asymmetric and composite membranes present the porous rigid frameworks with clear boundaries between the polymer mass and the pores. The shape and diameter of these pores can vary over a wide range and depend on the method and conditions of formation [6,7]. It can be seen (Figure 1) that in the transition from the surface layer to the porous one, the size of the pores changes relatively smoothly [8,9].

The driving force of mass transfer (gas permeation) through the membrane is the difference in the partial pressures of the mixture components in the high pressure and drainage channels [10,11,12]. Thus, the membrane itself during the separation process is under constant external pressure and is acted upon by tensile or compressive forces.

In industrial applications, composite and asymmetric membranes are used, and they can withstand high physicomechanical stresses. The porous layer of such membranes acts as a support. The optimal operating conditions of gas separation units require high values of permeate-specific flow; therefore, it is desirable to maintain a high-pressure difference of the separation process. In industrial applications, pressure difference between the high-pressure and drainage channels of the membrane module can reach relatively high values (50 and more atmospheres). Under such operating conditions, the membrane is naturally exposed to strong mechanical stresses [13,14].

Despite a large number of publications describing the mechanical properties of polymers and polymeric membranes, the effect of external pressure on the structure and, as a consequence, the separation characteristics of polymer membranes, remains unsolved [15,16].

It was shown in [16] that operating pressure causing deformation of the hollow fiber should not lead to plastic deformation in order to ensure that the transport characteristics of the membrane will remain unchanged.

Experimental measurement of the main mechanical characteristics (Young’s modulus, Poisson’s ratio, elongation and stress at break) remains the most important instrument for the study of hollow fiber membranes [16,17]. However, in the case of an anisotropic or multilayer structure, the experimentally-measured characteristics are averages; therefore, the data does not allow for the determination of the beginning of plastic deformation of the selective layer.

Using the finite element method in [18], the mechanical parameters (Young’s modulus, yield strength) of a microfiltration symmetric membrane were determined.

The method of reversible multiscale homogenization, which is used for numerical evaluation of the effective physical and mechanical characteristics of composite materials, is very promising for the study of the mechanical characteristics of hollow fiber membranes [19]. This method allows for the calculation of the characteristics of membranes with an anisotropic and multilayer structure. A significant disadvantage of this method is the high requirement for computing resources.

This paper presents a method for calculating the stress and deformation of hollow-fiber double-layer membranes based on the known data of the average characteristics of each of the layers. This allows us to take into account the differences in the onset of plastic deformation in the selective layer and the porous substrate and to determine the permissible operating pressure at which the selective layer will retain its characteristics.

## 2. Background

**Figure 2:**
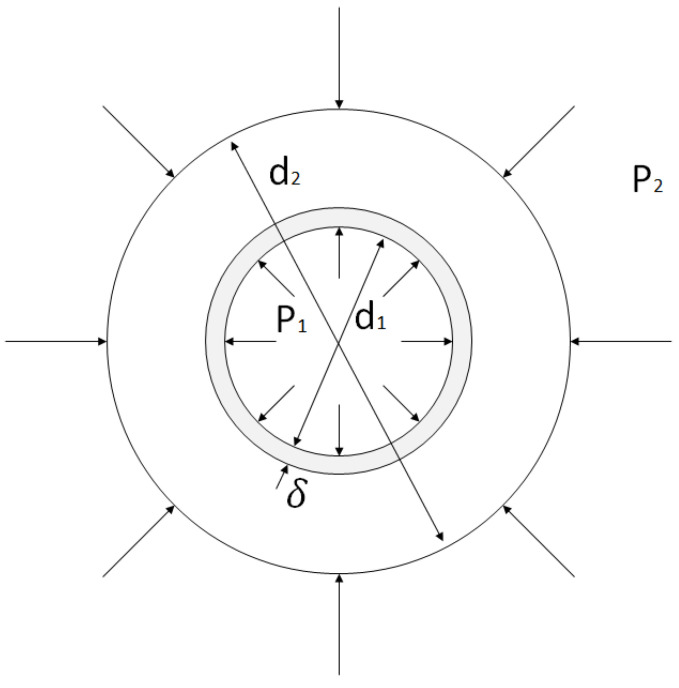
Model of a thick-walled cylinder under pressure.

Geometrically, a hollow fiber is a thick-walled concentric anisotropic cylinder, having at least two distinct layers: a porous substrate and a non-porous selective layer. Thus, the simplest model of a hollow fiber membrane element is a two-layer non-uniform hollow cylinder (Figure 2).

Figure 2 shows a simplified model of a hollow fiber membrane with a δ-thick selective layer located on the inner surface of the membrane. The inner cavity of the cylinder with a diameter $d_{1}$ forms the high-pressure channel of the membrane. The fiber outer diameter is $d_{2}$.

The calculations of the stress-strain state of a thick-walled isotropic cylinder were made by the French scientist G. Lamé [20,21]. The values of the stress and strain for the cylinder material (i.e., hollow fiber membrane element) could be obtained by solving an equation of the equilibrium:

(1) $$\frac{d\sigma_{r}}{dr}+\frac{\sigma_{r}+\sigma_{\theta }}{r}=0$$

where $\sigma_{r}$ is radial stress (MPa), $\sigma_{\theta }$ is circumferential stress (MPa), and r is cylinder radius (m).

Within the framework of the linear theory of elasticity, this equation can be rewritten with respect to the displacement vector (u), obtaining a linear differential equation of the second order:

(2) $$\frac{d^{2}u}{dr^{2}}+\frac{1}{r}\frac{du}{dr}-\frac{u}{r^{2}}=0$$

The general solution to this equation is as follows:

(3) $$u=C_{1}r+\frac{C_{2}}{r}$$

Integration constants ($C_{1}$ and $C_{2}$) could be found from the boundary conditions—the pressure values on the inner and outer surfaces of the hollow fiber [20].

To find the stress state of a two-layer cylinder, it is necessary to solve the conjugate problem for two concentric cylinders. In [21], the problem of “loading” a two-layer hollow cylinder with different mechanical characteristics of the layers was solved.

To obtain the distribution of mechanical stress along the radius of the cylinder and its deformation, it is necessary to know the physical and mechanical characteristics (Young’s modulus and Poisson’s ratio) of the inner and outer layers’ material (polymer).

Chen et al. [8] presented several physicomechanical characteristics of commercial polymers used for the production of hollow fiber membranes (Table 1).

It should be noted that the polymers under consideration have relatively high glass transition temperatures, and corresponding membrane materials under operating conditions are in a glassy state as well.

The advantage of using glassy polymers and membranes based on them is their relatively high physicomechanical and gas separation characteristics. High molecular-weight glassy polymers can withstand rather significant deformations [22].

**Figure 3:**
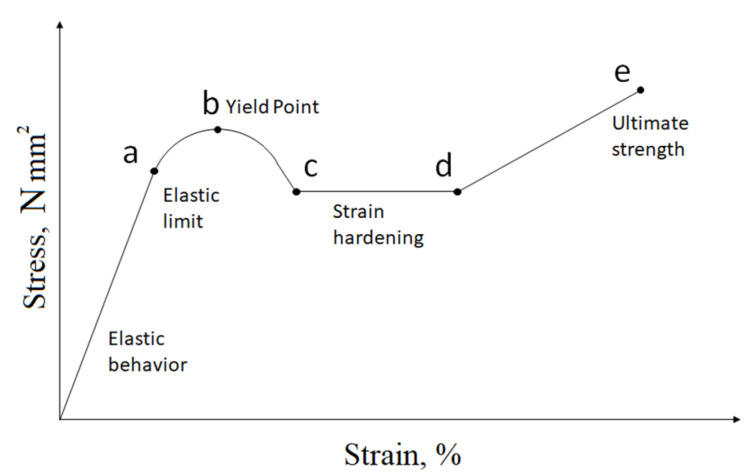
Dependence of strain on stress for glassy polymers [22].

Figure 3 shows a typical stress-strain curve for these polymers.

When high stresses are applied to a polymer sample and, consequently, to the corresponding polymeric membranes, forced elastic deformations in it arise, which cannot be removed at temperatures below the glass transition temperature [22]. In Figure 3, the development of “forced” elasticity is represented by the (a–b) segment.

From a practical (operational) point of view, the deformation of the sample should remain within the area (0–a)—a rectilinear section of reversible deformations, obeying Hooke’s law. The main parameters that characterize this section are the elastic limit and the Young’s modulus—the proportionality coefficient of the linear section.

Schulte K. et al. [23] studied the physicomechanical characteristics of polyimide (PI) and polyethersulfone (PES).

For example, from the dependence of deformation on stress given for PES, it can be concluded that in the interval of elastic deformations their relative elongation does not exceed 5%.

Asymmetric (or composite) membranes themselves present rather complicated structure with the predominant volume of the porous substrate.

The properties of porous material are influenced by the characteristics of the framework substance, the topology, and shape of the pores, as well as porosity.

**Figure 4:**
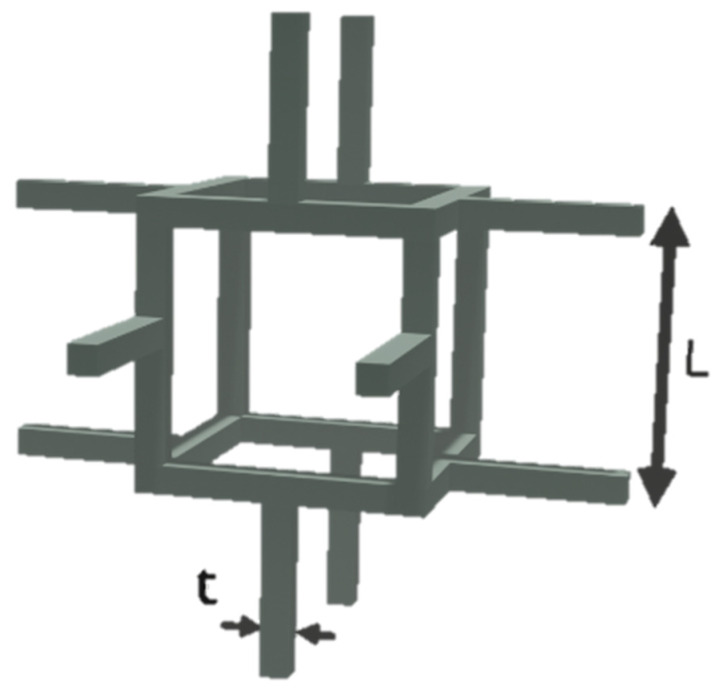
Unit cell model of a cellular porous material, Adapted from [24].

The porous layer of the polymer membrane has a structure which corresponds to the model of cellular porous material (Figure 4) [7,24].

The main structure characteristic of the cell in this model is its relative density:

(4) $$\frac{\tilde{\rho }}{\rho_{s}}\propto {\left(\frac{t}{L}\right)}^{2}$$

where $\tilde{\rho }$ is the density of the porous material, $\rho_{s}$ is the density of dense matter, L is the cell’s size, and t is the thickness of the cell’s edges.

The mechanical characteristics of such structures can be calculated using the method described by Gibson and Ashby [25].

According to their theory, Young’s modulus of a cellular porous body depends on the relative porosity and is expressed as:

(5) $$\frac{\tilde{E}}{E_{s}}\approx \frac{1}{3}{\left(\frac{\tilde{\rho }}{\rho_{s}}\right)}^{2}$$

where $\tilde{E}$ is Young’s modulus of porous material and $E_{s}$ is Young’s modulus of dense matter.

Therefore, with an increase in the porosity of the substrate, Young’s modulus decreases, which means that the material’s ability to resist the tension/compression phenomena decreases. Table 2 [8] presents some physicomechanical (stress-strain) characteristics of the hollow fiber membranes made from polymers presented in Table 1.

It can be seen that Young’s modulus of the polyethersulfone membrane is significantly lower than that of the polymer itself. Therefore, it could be concluded that the main contribution to the membrane’s strength is made by the support layer. As such, it could be assumed that the physicomechanical characteristics of the membranes (Table 2) coincide with the same characteristics for the support layer.

The calculations of the stress state of hollow fibers (two-layer cylinder) are similar to those made for a thick-walled cylinder. The general solution for each layer of a two-layer thick-walled cylinder will be as follows:

(6) $$u^{\left(1\right)}=C_{1}r+\frac{C_{2}}{r}$$

(7) $$u^{\left(2\right)}=C_{3}r+\frac{C_{4}}{r}$$

where $C_{1},\;C_{2},\;C_{3},\;C_{4}$ are integration constants.

Hooke’s Law for plane stress states:

(8) $$\sigma_{r}=\frac{E}{1-\mu^{2}}\left(\varepsilon_{r}+\mu \varepsilon_{\theta }\right)$$

(9) $$\sigma_{\theta }=\frac{E}{1-\mu^{2}}\left(\varepsilon_{\theta }+\mu \varepsilon_{r}\right)$$

where $\varepsilon_{r}=\frac{du}{dr}$, $\varepsilon_{\theta }=\frac{u}{r}$.

Substituting expressions (3) into (8)–(9), we obtain a solution for finding the stress at points located at a distance r from the cylinder axis:

(10) $$\sigma_{r}=\frac{E}{1-\mu^{2}}\left(C_{1}\left(1+\mu \right)-C_{2}\frac{1-\mu }{r^{2}}\right)$$

(11) $$\sigma_{\theta }=\frac{E}{1-\mu^{2}}\left(C_{1}\left(1+\mu \right)+C_{2}\frac{1-\mu }{r^{2}}\right)$$

Integration constants could be found from the boundary conditions (pressure on the outer and inner walls, displacement and stress of the boundary between layers), which give the following system of Equations (12)–(15):

(12) $$\sigma_{r}^{\left(1\right)}=\frac{E_{1}}{1-\mu_{1}^{2}}\left[C_{1}\left(1+\mu_{1}\right)-C_{2}\frac{1-\mu_{1}}{r_{1}^{2}}\right]=-P_{1}$$

(13) $$\sigma_{r}^{\left(2\right)}=\frac{E_{2}}{1-\mu_{2}^{2}}\left[C_{3}\left(1+\mu_{2}\right)-C_{4}\frac{1-\mu_{2}}{r_{2}^{2}}\right]=-P_{2}$$

(14) $$\frac{E_{1}}{1-\mu_{1}^{2}}\left[C_{1}\left(1+\mu_{1}\right)-C_{2}\frac{1-\mu_{1}}{r_{3}^{2}}\right]=\frac{E_{2}}{1-\mu_{2}^{2}}\left[C_{3}\left(1+\mu_{2}\right)-C_{4}\frac{1-\mu_{2}}{r_{3}^{2}}\right]$$

(15) $$C_{1}r_{c}+\frac{C_{2}}{r_{3}}=C_{3}r_{c}+\frac{C_{4}}{r_{3}}$$

where $\mu_{1},\;\mu_{2}$ are Poisson’s ratios for layers 1 and 2, respectively, $E_{1},\;E_{2}$ are Young’s modulus for layers 1 and 2, respectively, $p_{1},\;p_{2}$ represent the pressure inside and outside the fiber, $r_{1}$ is the fiber inner radius; $r_{2}$ is the fiber outer radius, and $r_{3}$ is the porous-selective layer boundary radius.

The stress state and deformation parameters for hollow fiber membranes (polymeric) could be obtained using the following data:

- The selective layer on the inner or outer surface of the fiber;
- The initial gas mixture which is fed into or outside the fiber;
- Inner diameter $d_{1}$= 100 microns;
- Selective layer thickness **δ** = 100 nm;
- Membrane material: Ultrason E6020P (PES);
- Young’s modulus for the selective layer and the porous substrate layer: $E_{1}=2650\;\mathrm{MPa}$, $E_{2}=72\;\mathrm{MPa}$, respectively;

- Poisson’s ratio for both layers equal to 0.35 [25].

## 3. Results and Discussion

**Figure 5:**
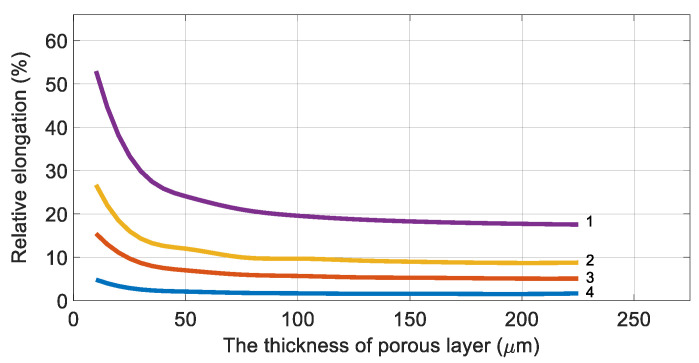
The relative elongation (ε) versus the thickness of the porous layer (h) at various pressures in the high-pressure channel: 1—10 MPa, 2—5 MPa, 3—3 MPa, and 4—1 MPa. The selective layer is located on the inner surface of the fiber.

Based on Equations (10)–(15), the dependences of the relative elongation of the selective layer versus the substrate thickness were obtained for various values of the working pressure (Figure 5).

It can be seen that as the thickness of the substrate increases, the relative elongation of the selective layer at constant pressure asymptotically decreases along the abscissa axis.

It can be seen as well that the fibers of the polyethersulfone (PES) membrane experience elastic deformation at the operating pressure not exceeding 3 MPa, and the thickness of the porous substrate must exceed the value of 100 µm.

At higher operating pressure, an increase in the porous substrate thickness will not lead to a rise in mechanical strength; therefore, for gas separation systems with high operating pressure, it is necessary to choose to use membranes made of polymers with better physico-mechanical characteristics.

When a selective layer is formed on the outer surface of the membrane, the pressure channel in the corresponding module is the space between the fibers, and, consequently, the hollow fibers are subjected to compression.

For plastic materials, the numerical values of the yield stress in compression and tension are approximately equal [26], as well as Young’s modulus; therefore, the calculation can be carried out using the same system of equations and the values of elastic constants.

**Figure 6:**
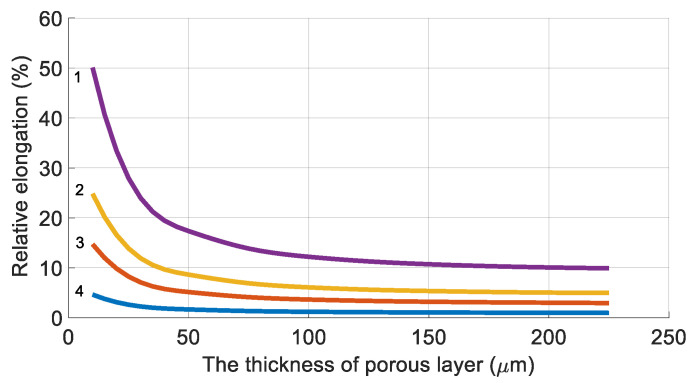
The relative elongation (ε) versus the thickness of the porous layer at various pressures in the high-pressure channel: 1—10 MPa, 2—5 MPa, 3—3 MPa, and 4—1 MPa.

Figure 6 shows the dependences of the relative circumferential elongation on the thickness of the porous layer (substrate) with an inner fiber diameter of 100 μm for membranes with a selective layer on the outer surface.

Therefore, it could be concluded that (Figure 5 and Figure 6) the nature of the dependence of the selective layer’s relative elongation on the thickness of the substrate is retained.

The results obtained show that membranes with an outer selective layer can experience relatively heavy loads. The maximum operational pressure, at which the elongation does not exceed 5% for the selected polymer, can reach values up to 5 MPa.

**Figure 7:**
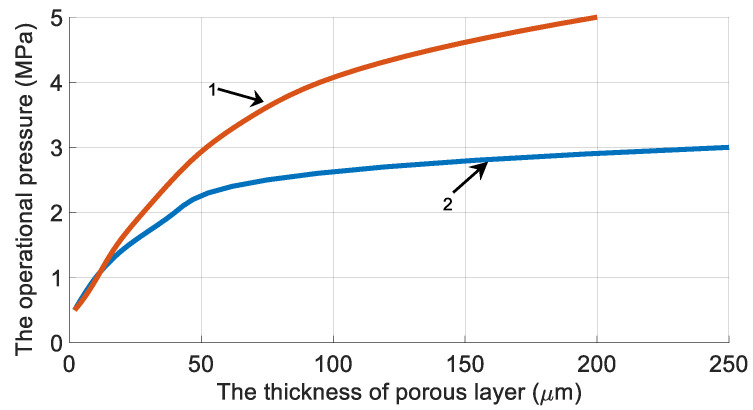
The influence of the porous layer thickness (h) on the applied operational pressure $\left(P_{max}\right)$ selective layer outside (1) and inside (2) the fiber.

Figure 7 shows the dependencies of the maximum operating pressure (at higher pressure, plastic deformation occurs—the relative elongation of the selective layer is more than 5%) on the thickness of the porous membrane layer with an inner diameter of 100 μm for both cases of selective layer deposition (outside and inside the fiber).

Thus, fibers with an outer membrane selective layer allow a higher operational pressure to be used.

## 4. Conclusions

This paper presents the simulation of permissible loads (operational pressure) on a membrane in the form of a hollow fiber.

The stress states and deformations of hollow fibers are calculated at different operating pressures and substrate thicknesses. It is shown that the dependence of the maximum allowable operating pressure on the substrate thickness asymptotically trends to the maximum pressure value for a given membrane. Thus, a further increase in the thickness of the substrate will not allow it to withstand higher operation pressures.

These calculations were performed for two types of hollow fiber membranes with a selective layer inside and outside the fiber. Fibers with an outer selective layer can withstand higher pressures, which makes this configuration more advantageous.

Despite several significant advantages of the outer selective layer deposition, the optimal parameters for hollow fiber membrane gas separation systems should be realized solving the optimization problem and taking into account:

- the physico-chemical nature of the membrane polymer;
- physico-chemical properties (morphology, structure, permeability, selectivity) of the skin layer;
- physico-mechanical properties (Young’s modulus, elastic limit, etc.) of membrane layers;
- the porosity of the membrane support layer;
- hollow fiber dimensions—outer and inner diameters, length, and their influence on each other.

## Figures and Tables

**Table 1:** Physical and mechanical characteristics of polymers [8].

Materials	Glass Transition Temperature (°C)	Young’s Modulus, (MPa)	Elongation at Break (%)	Tensile Strength (MPa)
Ultem^@^ 1000 (PEI)	217	3585	60	105
Matrimid^@^ 5218 (PI)	319	2896	49	86.9
Ultrason E6020P (PES)	225	2650	50–100	85

**Table 2:** Physical and mechanical characteristics of membranes.

Membranes (Materials)	Young’s Modulus (MPa)	Elongation at Break (%)	Ultimate Strength (MPa)	Porosity (%)
U305 (Ultem^@^ 1000 (PEI))	132	44	58.5	55.9
M264 (Matrimid^@^ 5218 (PI))	121	29	54.8	58.4
PES28 (Ultrason E6020P (PES))	72	85	5.2	46.1

## Data Availability

Not applicable.

## Nomenclature

**Symbol**	**Description**
$d_{1}$	inner fiber diameter
$d_{2}$	outer fiber diameter
$P_{1}$	pressure inside the fiber
$P_{2}$	pressure outside the fiber
δ	selective layer thickness
$\sigma_{r}$	radial stress
$\sigma_{\theta }$	circumferential stress
r	cylinder radius
u	displacement vector
C	integration constant
ε	relative elongation (strain)
σ	stress
E	Young’s modulus
μ	Poisson’s ratio
$\varepsilon_{r}$	radial strain
$\varepsilon_{\theta }$	circumferential strain
$\tilde{\rho }$	the density of the porous material
$\rho_{s}$	density of dense matter
L	cell size
t	the thickness of the cell’s edges
$\tilde{E}$	Young’s modulus of porous material
$E_{s}$	Young’s modulus of dense matter
